# Eugenol Suppresses Platelet Activation and Mitigates Pulmonary Thromboembolism in Humans and Murine Models

**DOI:** 10.3390/ijms25042098

**Published:** 2024-02-08

**Authors:** Wei-Chieh Huang, Lan-Hsin Shu, Yu-Ju Kuo, Kevin Shu-Leung Lai, Chih-Wei Hsia, Ting-Lin Yen, Chih-Hsuan Hsia, Thanasekaran Jayakumar, Chih-Hao Yang, Joen-Rong Sheu

**Affiliations:** 1 Graduate Institute of Medical Sciences, College of Medicine, Taipei Medical University, Taipei 110, Taiwan23580@s.tmu.edu.tw (Y.-J.K.); 2 Graduate Institute of Pharmacology, College of Medicine, National Taiwan University, Taipei 106, Taiwan; 3 Division of Critical Care Medicine, Taipei Medical University Hospital, Taipei 110, Taiwan; 4 Department of Medical Research, Taipei Medical University Hospital, Taipei 110, Taiwan; 5 Department of Medical Research, Cathay General Hospital, Taipei 106, Taiwan; 6 Translational Medicine Center, Shin Kong Wu Ho-Su Memorial Hospital, Taipei, 111, Taiwan; 7 Department of Ecology and Environmental Sciences, Pondicherry University, Puducherry 605014, India; jayakumar@tmu.edu.tw; 8 Department of Pharmacology, School of Medicine, College of Medicine, Taipei Medical University, Taipei 110, Taiwan

**Keywords:** eugenol, PLCγ2–PKC, cPLA2/TxA_2_, MAPK, human platelets, pulmonary thrombosis, PI3K/Akt/GSK-3β

## Abstract

Platelets assume a pivotal role in the pathogenesis of cardiovascular diseases (CVDs), emphasizing their significance in disease progression. Consequently, addressing CVDs necessitates a targeted approach focused on mitigating platelet activation. Eugenol, predominantly derived from clove oil, is recognized for its antibacterial, anticancer, and anti-inflammatory properties, rendering it a valuable medicinal agent. This investigation delves into the intricate mechanisms through which eugenol influences human platelets. At a low concentration of 2 μM, eugenol demonstrates inhibition of collagen and arachidonic acid (AA)-induced platelet aggregation. Notably, thrombin and U46619 remain unaffected by eugenol. Its modulatory effects extend to ATP release, P-selectin expression, and intracellular calcium levels ([Ca^2+^]i). Eugenol significantly inhibits various signaling cascades, including phospholipase Cγ2 (PLCγ2)/protein kinase C (PKC), phosphoinositide 3-kinase/Akt/glycogen synthase kinase-3β, mitogen-activated protein kinases, and cytosolic phospholipase A2 (cPLA2)/thromboxane A2 (TxA_2_) formation induced by collagen. Eugenol selectively inhibited cPLA2/TxA_2_ phosphorylation induced by AA, not affecting p38 MAPK. In ADP-treated mice, eugenol reduced occluded lung vessels by platelet thrombi without extending bleeding time. In conclusion, eugenol exerts a potent inhibitory effect on platelet activation, achieved through the inhibition of the PLCγ2–PKC and cPLA2-TxA_2_ cascade, consequently suppressing platelet aggregation. These findings underscore the potential therapeutic applications of eugenol in CVDs.

## 1. Introduction

Cardiovascular diseases (CVDs) are a major global health challenge and a leading cause of mortality. Central to CVD pathogenesis is arterial thrombosis, serving as the primary initiator. Platelets, crucial for hemostasis, contribute to vascular injury recovery. However, hyperactivated platelets, influenced by various pathophysiological factors, lead to complications in arterial thrombosis. This sequence significantly contributes to atherosclerosis, thrombosis, coronary heart disease, stroke, and myocardial infarction [1].

Platelets, derived from megakaryocytes [2], typically remain quiescent under normal conditions but become activated during intraluminal thrombosis. Activation involves processes like adherence and aggregation, triggered when a blood vessel is injured. Platelets lack spontaneous aggregation in circulation without vascular damage. Upon injury, platelets adhere to the disrupted vascular surface, releasing biologically active substances and aggregating [3]. Collagen plays a pivotal role in platelet adhesion and activation through interactions with collagen receptors. This cascade results in the release of adenosine diphosphate (ADP) and the synthesis of thromboxane A_2_ (TxA_2_).

**Figure 1:**
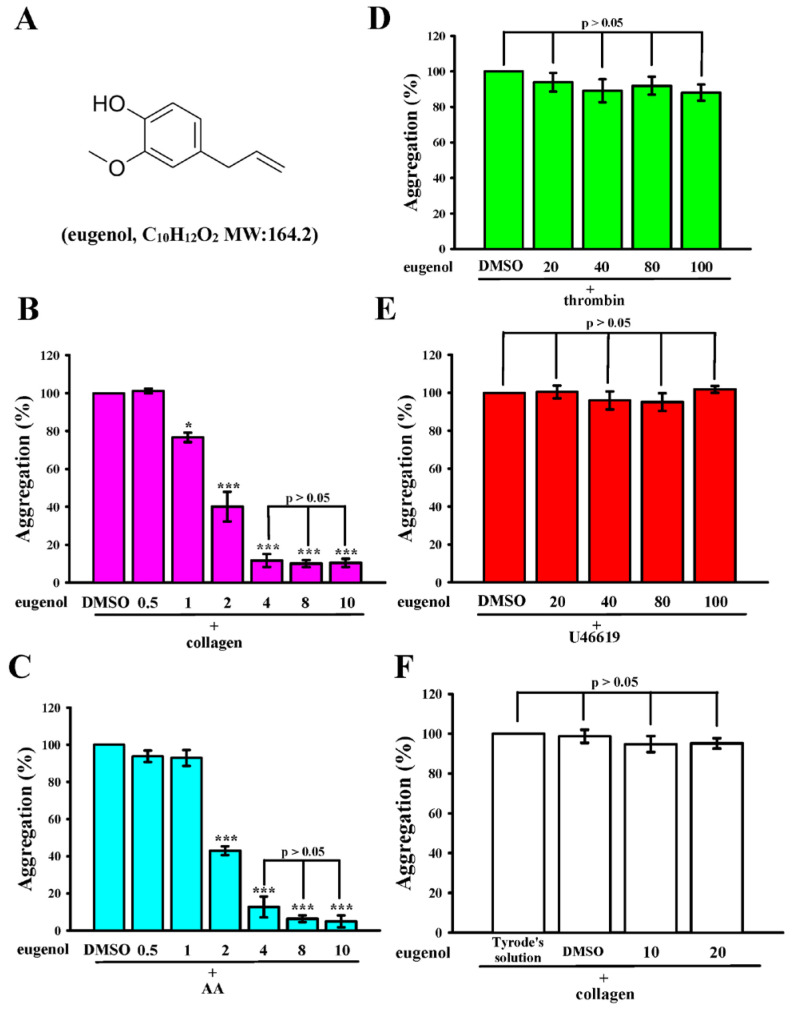
(**A**) The molecular structure of eugenol, with the molecular formula C_10_H_12_O_2_, is illustrated. Washed human platelets (3.6 × 10^8^ cells/mL) were preincubated with either a solvent control (0.1% DMSO) or varying concentrations of eugenol (0.5 to 100 μM). Subsequently, platelets were exposed to different agonists, including (**B**) collagen (1 μg/mL), (**C**) arachidonic acid (AA; 60 μM), (**D**) thrombin (0.02 U/mL), or (**E**) U46619 (1 μM), to induce platelet aggregation. Concentration-response histograms for eugenol highlight its inhibitory effects on platelet aggregation triggered by various agonists (%). (**F**) Cytotoxicity assessment involved preincubating platelets with 0.1% DMSO, 10 μM, or 20 μM eugenol for 10 min, followed by two washes with Tyrode’s solution. Subsequently, platelets were stimulated with collagen (1 μg/mL). Statistical significance levels of * *p* < 0.05 and *** *p* < 0.001 indicate differences compared to the 0.1% DMSO-treated group. The presented data in (**B**–**F**) represent the mean ± standard error of the mean (*n* = 4).

Eugenol (C_10_H_12_O_2_), a phenylpropanoid illustrated in Figure 1A, represents an aromatic compound within the phenol group. It is derived from the natural essential oils of plants, prominently found in cloves (*Syzygium aromaticum*), boasting a rich historical tradition of use. Acknowledged for its diverse pharmacological activities, including analgesic, anti-inflammatory, antioxidant, vasodilation, and potential anticancer effects [4]. Widely employed as a painkiller and anesthetic in dental practice, eugenol has been observed to inhibit voltage-gated sodium channels in dental neuron studies [5,6]. Notably, its antibacterial efficacy extends to various species, such as Gram-positive and negative bacteria. Mechanistically, eugenol is presumed to exert its antibacterial effects by inducing damage to the cytoplasmic membrane, facilitated by its ability to readily penetrate the bacterial cell membrane and access the cytoplasm [4,7,8].

Antiplatelet drugs, including integrin α_IIb_β_3_ antagonists, aspirin, and clopidogrel, are designed to prevent excessive platelet activation [9,10,11]. However, their efficacy is often hindered by side effects, like aspirin-induced gastric ulcers and bleeding, and clopidogrel-associated issues such as aplastic anemia and thrombocytopenic purpura [12,13]. There is a critical need for novel agents with improved safety and efficacy in treating and preventing cardiovascular diseases, ideally with minimal or no drug-related complications.

Eugenol has exhibited diverse and notable pharmacological activities; however, there is limited research addressing the specific impact of eugenol on platelet activation, particularly its role in humans. Only a solitary study has underscored the potency of eugenol, surpassing that of aspirin, in inhibiting human platelet aggregation induced by arachidonic acid (AA) [14]. Consequently, the current study is dedicated to investigating the antiplatelet effects of eugenol in human subjects and assessing its therapeutic efficacy through an in vivo model.

## 2. Results

### 2.1. Eugenol Inhibits Collagen and AA-Induced Platelet Aggregation in Humans

Eugenol, as illustrated in Figure 1A, displayed notable efficacy in inhibiting platelet aggregation induced by both collagen (1 μg/mL) and AA (60 µM) within the concentration range of 1 to 4 μM (Figure 1B,C). Remarkably, even at concentrations as high as 100 μM, eugenol exhibited no significant effects when stimulated by thrombin (0.02 U/mL) or 11-dideoxy-11α,9α-epoxymethanoprostaglandin (U46619, 1 µM), a prostaglandin endoperoxide (Figure 1D,E). In addition, eugenol at concentrations of 40 and 100 µM did not exert a significant inhibitory effect on the stimulation induced by thrombin (0.01 U/mL) and U46619 (0.5 µM) in washed human platelets (Figure S1). The calculated IC_50_ of eugenol was approximately 2 μM for both collagen and AA stimulation. Subsequently, the IC_50_ value (2 μM) and the maximal concentration (4 µM) of eugenol were employed to delve into the potential mechanisms underlying its inhibitory activity on platelet activation. Notably, platelets preincubated with 0.1% DMSO, or 10 and 20 μM eugenol for 20 min, and subsequently washed two times with Tyrode’s solution, exhibited aggregation curves insignificantly different from those of platelets preincubated with the solvent control (Tyrode’s solution) under equivalent conditions (Figure 1F). This observation suggests that the effects of eugenol on platelet aggregation are reversible and noncytotoxic. Furthermore, lactate dehydrogenase (LDH) assay results demonstrated that treatment with eugenol (10–100 μM) did not induce any notable release of LDH when platelets were pre-treated for 20 min (Figure S2). This finding indicates that eugenol exhibited negligible cytotoxicity towards platelets.

### 2.2. Eugenol Modulates ATP Release, [Ca^2+^]i Levels, and P-Selectin Surface Expression

**Figure 2:**
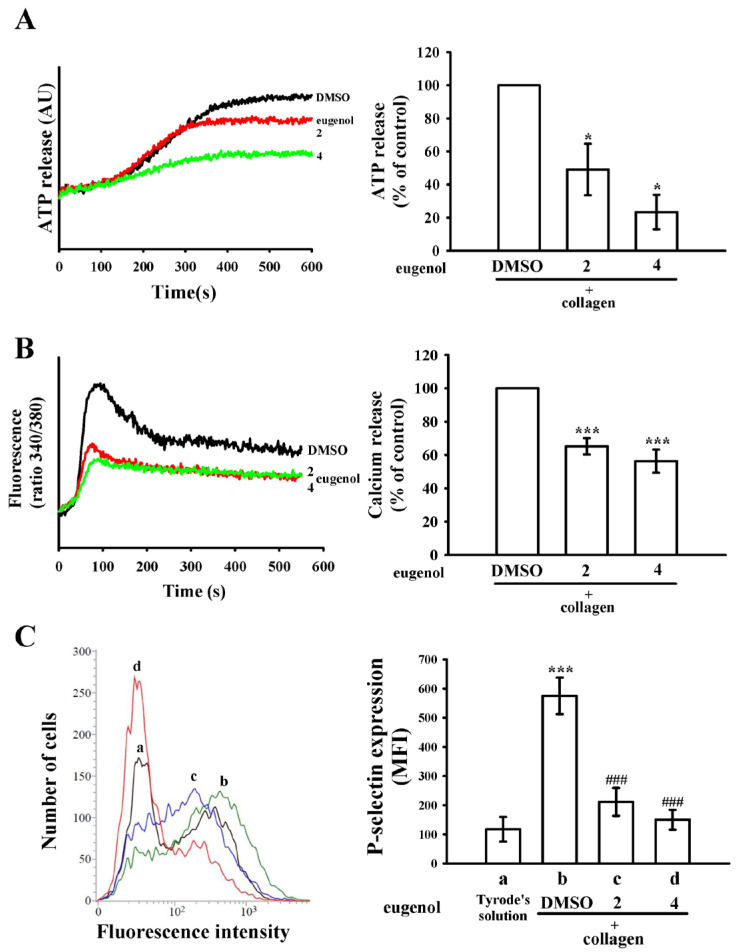
Eugenol’s inhibitory effects on ATP release, relative [Ca^2+^]i level, and surface P-selectin expression in human platelets were investigated. Washed platelets (3.6 × 10^8^ cells/mL) were preincubated with either 0.1% DMSO or eugenol (2 and 4 µM), followed by collagen (1 μg/mL) stimulation to elicit the following responses: (**A**) ATP release, quantified in arbitrary units (AU); (**B**) relative [Ca^2+^]i level; and (**C**) surface P-selectin expression (a, Tyrode’s solution; b, 0.1% DMSO + collagen group; c, 2 µM eugenol + collagen group; d, 4 µM eugenol + collagen group). Detailed experimental methodologies are provided in Section 4. Statistical significance in (**A**,**B**) is denoted by * *p* < 0.05 and *** *p* < 0.001 compared to the 0.1% DMSO-treated group. In (**C**), *** *p* < 0.001 indicates deviations from the resting control (Tyrode’s solution), while ^###^ *p* < 0.001 signifies differences compared to the 0.1% DMSO-treated group. The data are presented as the mean ± standard error of the mean (*n* = 4).

Eugenol, when administered at concentrations of 2 and 4 µM, exhibited a concentration-dependent reduction in collagen-induced ATP release, as depicted in Figure 2A. Moreover, both concentrations of eugenol demonstrated significant attenuation of the elevation in intracellular calcium ([Ca^2+^]i) levels induced by collagen, with reductions of approximately 35% and 44%, respectively (Figure 2B). P-selectin, a pivotal biomarker for platelet activation, typically resides within the inner walls of α-granules. Upon activation, platelets unveil the inner granule contents to the outer membrane [15]. In Figure 2C, the suppressive effect of eugenol on collagen-stimulated surface FITC-P-selectin expression is illustrated (a, Tyrode’s solution, 117 ± 42; b, 0.1% DMSO + collagen group, 575 ± 63; c, 2 µM eugenol + collagen group, 212 ± 48; d, 4 µM eugenol + collagen group, 150 ± 34; *n* = 4). Detailed statistical data are provided in the right-hand panels of Figure 2.

### 2.3. Evaluating Eugenol’s Impact on Platelet cPLA2, PLCγ2 Phosphorylation, and PKC Activation

**Figure 3:**
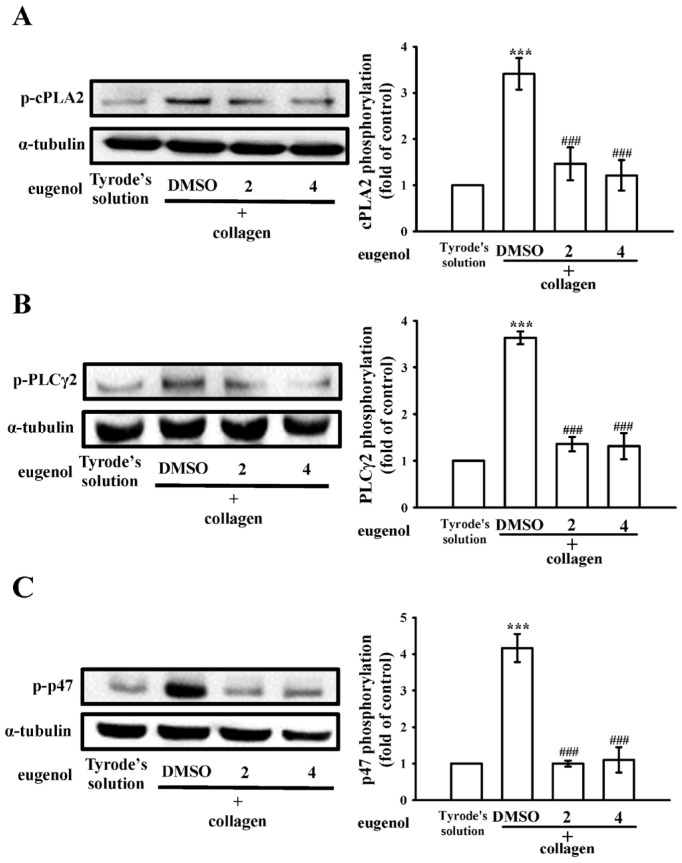
The impact of eugenol on the activation of cytosolic phospholipase A2 (cPLA2), phospholipase Cγ2 (PLCγ2), and protein kinase C (PKC) in platelets was investigated. Washed platelets were preincubated with either 0.1% DMSO or eugenol (2 and 4 µM) and subsequently stimulated with collagen (1 µg/mL) to induce the following responses: phosphorylation of (**A**) cPLA2, (**B**) PLCγ2, and (**C**) activation of PKC, as indicated by p-p47 phosphorylation. Data are presented as the mean ± standard error of the mean (*n* = 4). Significant differences are denoted by *** *p* < 0.001 in comparison to resting platelets exposed to Tyrode’s solution. Furthermore, ^###^ *p* < 0.001 is used to indicate disparities compared to the group treated with 0.1% DMSO.

**Figure 4:**
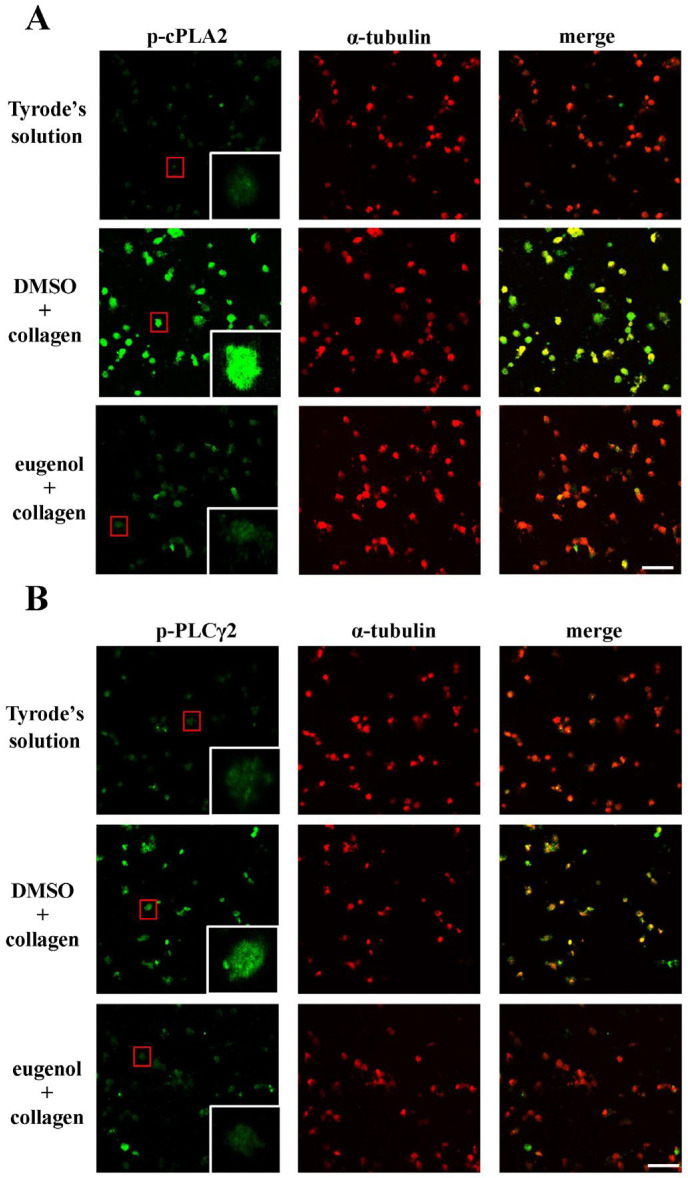
Eugenol’s inhibitory effects on the activation of cytosolic phospholipase A2 (cPLA2) and phospholipase Cγ2 (PLCγ2) were visualized using confocal laser microscopy. Washed platelets were pre-incubated with either 0.1% DMSO or eugenol (4 µM) and subsequently exposed to collagen (1 μg/mL) for confocal microscopic evaluation at 1000× magnification. This assessment specifically focused on the visualization of phosphorylated (**A**) cPLA2 and (**B**) PLCγ2, represented by green fluorescence, along with α-tubulin indicated by red fluorescence. The presented images are representative of four independent experiments. The red boxes serve to highlight one of the numerous cells that have been phosphorylated and are further magnified within white boxes. The scale bar is 10 μm.

Cytosolic phospholipase A2 (cPLA2) is a key enzyme in platelet activation, as its activation initiates the release of AA, which promotes platelet aggregation [16]. Concurrently, phospholipase C (PLC) participates in the intricate signaling cascade by hydrolyzing phosphatidylinositol 4,5-bisphosphate, generating two crucial secondary messengers—diacylglycerol (DAG) and inositol trisphosphate (IP_3_). DAG activates protein kinase C (PKC), leading to the phosphorylation of a predominantly 47-kDa protein (pleckstrin or p47) and subsequent granule secretion, while IP_3_ triggers calcium release from the dense tubular system [17]. In the context of our study, eugenol, administered at concentrations of 2 and 4 µM, exhibited a remarkable diminishment in cPLA2 and PLCγ2 phosphorylation as well as PKC activation (p-p47) in collagen-activated platelets (Figure 3). Further insights into the impact of eugenol on cPLA2 and PLCγ2 phosphorylation were gained through confocal scanning fluorescence microscopy, revealing green fluorescence indicative of cPLA2 or PLCγ2 activation, and red fluorescence representing α-tubulin in resting or collagen-activated platelets (Figure 4A,B). Collagen-induced fluorescence intensity of phosphorylated cPLA2 or PLCγ2 was notably reduced in eugenol (4 µM)-treated platelets, whereas α-tubulin intensity remained unchanged between groups (Figure 4). These findings collectively suggest that eugenol exerts its antiplatelet activity by effectively inhibiting cPLA2 and PLCγ2/PKC activation.

### 2.4. Regulatory Effects of Eugenol on PI3K-Akt-GSK3β and MAPKs Activation

**Figure 5:**
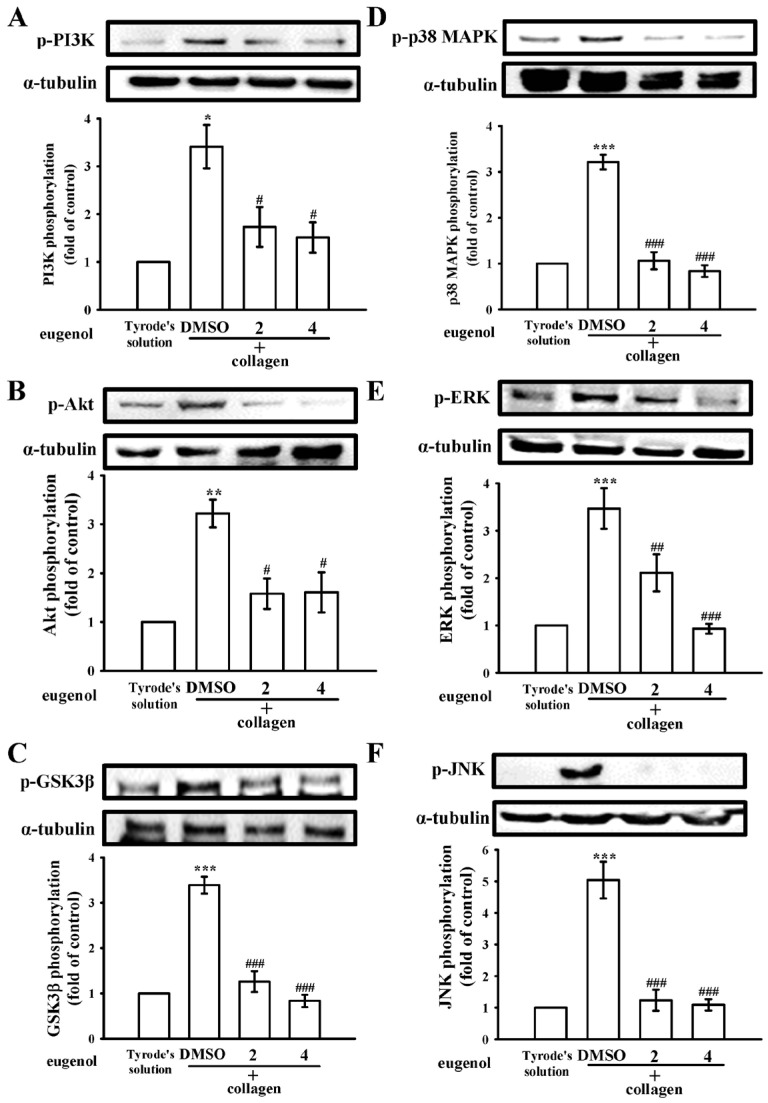
Illustrates the regulatory impact of eugenol on the phosphoinositide 3-kinase (PI3K)/Akt/glycogen synthase kinase-3β (GSK3β) and mitogen-activated protein kinases (MAPKs) pathways. Platelets were preincubated with either 0.1% DMSO or eugenol (2 and 4 µM) and subsequently exposed to collagen (1 μg/mL). This allowed for immunoblotting analysis of key components within the (**A**) PI3K, (**B**) Akt, (**C**) GSK3β, (**D**) p38 MAPK, (**E**) ERK, and (**F**) JNK pathways. The data are presented as the mean ± standard error of the mean (*n* = 4). Statistical significance is denoted as * *p* < 0.05, ** *p* < 0.01, and *** *p* < 0.001 compared with the results observed in resting platelets (Tyrode’s solution); and ^#^ *p* < 0.05, ^##^ *p* < 0.01, and ^###^ *p* < 0.001 compared with the results observed in the 0.1% DMSO group.

The phosphoinositide 3-kinase (PI3K)/Akt/ glycogen synthase kinase 3β (GSK3β) signaling pathway emerges as a pivotal player in thrombus formation under conditions of heightened shear stress [18]. Within this cascade, PI3K assumes a crucial role in orchestrating the activation of Akt, the principal regulatory node in the pathway [18]. Activation of the Akt pathway, triggered by various platelet agonists governing platelet activation and hemostasis, underscores its significance in platelet function. Moreover, GSK3β, a key factor, is subject to regulation by the PI3K/Akt pathway in platelets [19]. We observed that eugenol (2 and 4 µM) effectively curtailed the activation of the PI3K/Akt/GSK3β pathway in platelets upon collagen stimulation (Figure 5A–C). Furthermore, the mitogen-activated protein kinases (MAPKs) signaling pathways, encompassing p38 MAPK, extracellular signal-regulated kinase (ERK), and c-Jun N-terminal kinase (JNK), stands as a critical regulator of inflammation, cell proliferation, apoptosis, and platelet activation [20]. Remarkably, our findings demonstrate that eugenol exerted a suppressive effect on the phosphorylation of all three MAPKs induced by collagen. This observation suggests that the antiplatelet activation mediated by eugenol involves a marked modulation of MAPK pathways (Figure 5D–F). These findings collectively underscore the multifaceted impact of eugenol on key signaling pathways, shedding light on its potential therapeutic relevance in the intricate landscape of platelet function and thrombotic events.

### 2.5. Interplay of p38 MAPK, cPLA2, and TxA_2_ in Eugenol’s Antiplatelet Mechanism

**Figure 6:**
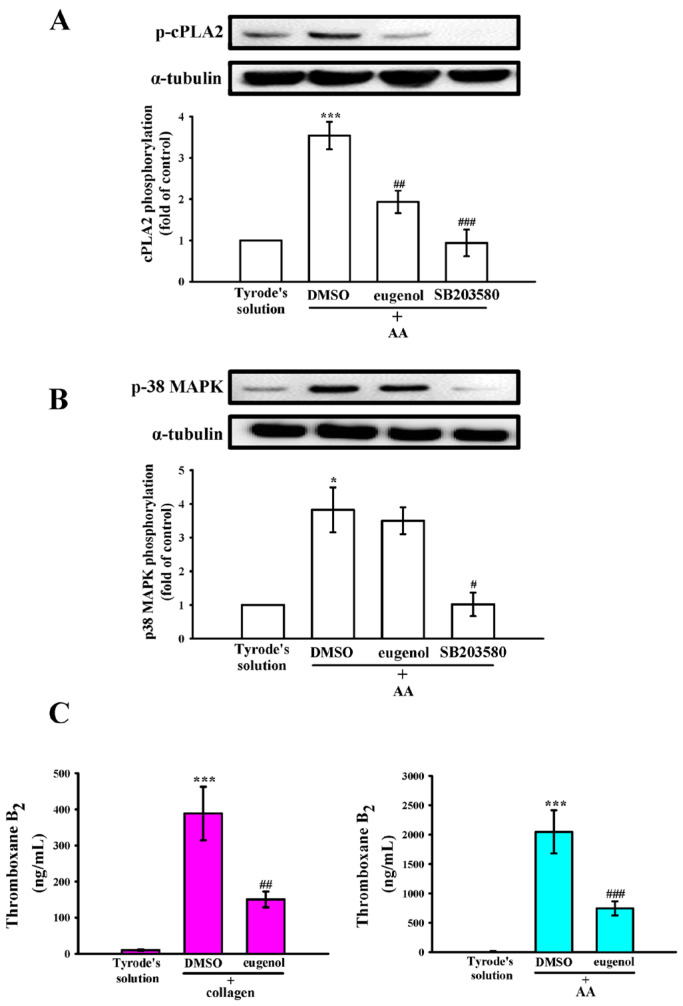
The study investigated the effects of eugenol on cytosolic phospholipase A2 (cPLA2) and p38 mitogen-activated protein kinase (p38 MAPK) phosphorylation, as well as thromboxane B_2_ (TxB_2_) formation in human platelets. Platelets were preincubated with either 0.1% DMSO, eugenol (4 µM) or with SB203580 (20 µM) and subsequently exposed to arachidonic acid (AA; 60 µM). Immunoblotting analysis was conducted to assess the levels of (**A**) cPLA2 and (**B**) p38 MAPK proteins. (**C**) In another set of experiments, platelets were preincubated with Tyrode’s solution alone, or with either 0.1% DMSO or eugenol (4 µM), followed by exposure to collagen (1 μg/mL) and AA (60 µM) to quantify TxB_2_ formation. The data are presented as the mean ± standard error of the mean (*n* = 4). Statistical significance is indicated as * *p* < 0.05 and *** *p* < 0.001 compared to the results observed in resting platelets (Tyrode’s solution); ^#^ *p* < 0.05, ^##^ *p* < 0.01 and ^###^ *p* < 0.001 compared to the results observed in the 0.1% DMSO group.

In Figure 6A,B, a conspicuous elevation in cPLA2 phosphorylation was also observed in response to AA (60 µM). Both eugenol (4 µM) and the p38 MAPK inhibitor SB203580 (20 µM) demonstrated a significant reduction in cPLA2 phosphorylation induced by AA. Interestingly, eugenol (4 µM) showed no effects on AA-stimulated p38 MAPK phosphorylation (Figure 6B). Moreover, AA assumes a pivotal role as a precursor in the biosynthesis of diverse bioactive lipid mediators, notably including TxA_2_, which recognized for its potency as both a platelet agonist and vasoconstrictor, intricately contributes to the augmentation of platelet activation and aggregation [21]. As delineated in Figure 6C, the levels of thromboxane B_2_ (TxB_2_) exhibited a conspicuous increase upon stimulation by collagen and AA. Notably, this elevation was significantly attenuated in the presence of eugenol (4 µM), indicating an inhibitory role of eugenol in the cPLA2-TxA_2_ pathway stimulated by collagen and AA.

### 2.6. Anti-Thrombotic Efficacy of Eugenol in Acute Pulmonary Thromboembolism in Mice

**Figure 7:**
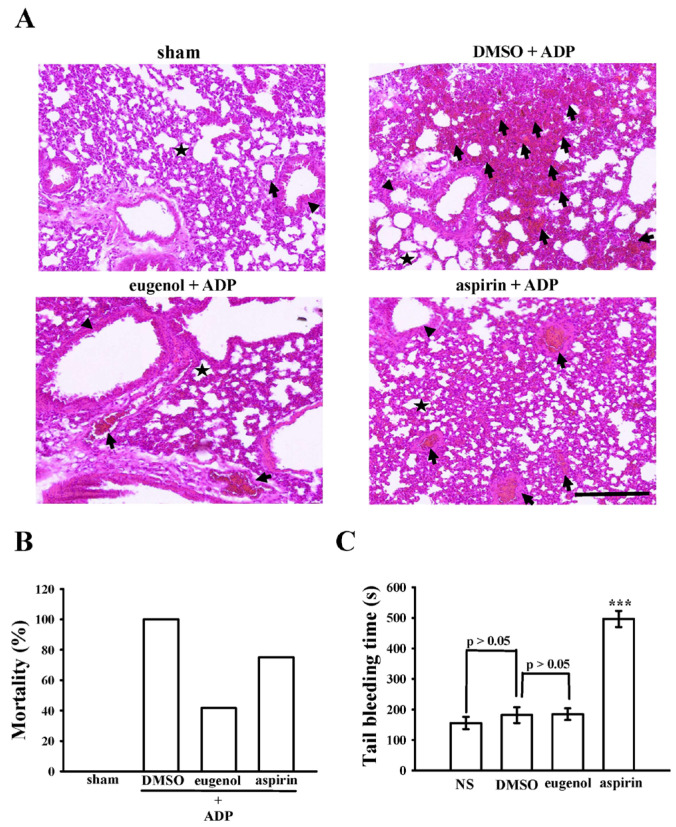
The study evaluated the efficacy of eugenol in mitigating thromboembolism in the lungs of mice. (**A**) Acute pulmonary thrombosis was induced by intraperitoneally administering either 0.1% DMSO, eugenol (15 mg/kg), or aspirin (15 mg/kg) to mice, followed by the injection of ADP (700 mg/kg) into the tail vein. Histological examination of lung tissue sections stained with hematoxylin–eosin focused on alveoli (stars), blood vessels (arrows), and bronchioles (arrowheads), with a scale bar indicating 200 μm. The mortality rate (%) of ADP-induced pulmonary thromboembolism in mice (*n*= 12) was presented in (**B**). (**C**) In a separate investigation, bleeding time was determined by transecting mouse tails after a 30-min interval following intraperitoneal administration of either normal saline (NS), 0.1% DMSO, eugenol (15 mg/kg), or aspirin (15 mg/kg). The data are presented as the mean ± standard error of the mean (*n* = 12). Statistical significance is denoted as *** *p* < 0.001 compared to the results observed in the 0.1% DMSO group.

Intravenous administration of ADP in murine subjects elicited an acute, platelet-dependent pulmonary thromboembolic response, manifesting as an increased mortality rate attributed to the occlusion of pulmonary vessels by platelet thromboemboli. Alveoli, integral air sacs facilitating oxygen and carbon dioxide exchange within the lungs (depicted as stars in Figure 7A), play a pivotal role in maintaining respiratory function. The blood vessels (indicated by arrows) surrounding the alveoli, along with bronchioles (arrowheads), conduits for air during breathing, constitute crucial components of the pulmonary microenvironment. Figure 7A illustrates a significantly elevated occurrence of completely or partially occluded lung vessels by platelet thrombi (arrows) in ADP-treated mice compared to the sham group. Treatment with eugenol at 15 mg/kg effectively reduced the number of occluded vessels in comparison to 0.1% DMSO treatment. Furthermore, both eugenol and aspirin treatments (15 mg/kg) demonstrated a notable reduction in occluded vessels compared to the 0.1% DMSO treatment. Moreover, eugenol and aspirin (15 mg/kg) substantially diminished the mortality rate from 100% (12 deceased, *n* = 12; 0.1% DMSO-treated group) to 41.6% (5 deceased, *n* = 12; *p* < 0.05) and 75% (9 deceased, *n* = 12; *p* < 0.05), respectively (Figure 7B). Bleeding time assessment, conducted by tail vein transection 30 min after intraperitoneal administration of aspirin and eugenol (15 mg/kg; Figure 7C), revealed non-significant changes in bleeding time between the normal saline (NS) group (156 ± 20 s; *n* = 12), solvent control group (0.1% DMSO, 182 ± 26 s; *n* = 12), and eugenol group (15 mg/kg; 185 ± 19 s; *n* = 12). In contrast, bleeding time markedly prolonged after 15 mg/kg aspirin treatment (497 ± 27 s; *n* = 12). To discern rebleeding tendencies, individual mice were monitored for 15 min even after cessation of bleeding. The findings suggest that eugenol exhibits in vivo antithrombotic activity without significantly impacting bleeding time at the effective dose of 15 mg/kg.

## 3. Discussion

Eugenol, an aromatic phenolic compound predominantly derived from clove oil, has enjoyed historical applications in diverse fields, including cosmetology, medicine, and pharmacology. The current study underscores the remarkable antiplatelet efficacy of eugenol, substantiated through both human and animal experimentation. In particular, our findings demonstrate that concentrations as modest as 4 µM of eugenol suffice to impede platelet activation induced by collagen. Notwithstanding that eugenol, when sourced from natural reservoirs, may fall short of attaining the requisite plasma concentrations for inhibition of in vivo platelet activation, its protracted consumption presents an advantageous strategy for averting atherothrombotic events. In light of these revelations, eugenol emerges as a compelling prospect for pioneering antithrombotic interventions in human subjects, given its conspicuously robust antiplatelet attributes.

Platelet activation instigates a complex array of tyrosine kinase cascades, culminating in heightened intracellular calcium concentrations and the exocytosis of granules containing notable constituents such as P-selectin and ADP/ATP. The principal repository for protein storage within platelets is dominated by α-granules, encompassing both membrane-associated proteins like P-selectin and various soluble proteins including fibrinogen and platelet-derived growth factor. The exocytosis-mediated release of α-granules stands as a pivotal hallmark of platelet activation. This activation status can be effectively gauged through the meticulous examination of P-selectin expression, a key molecular indicator, as elucidated in Figure 2C.

**Figure 8:**
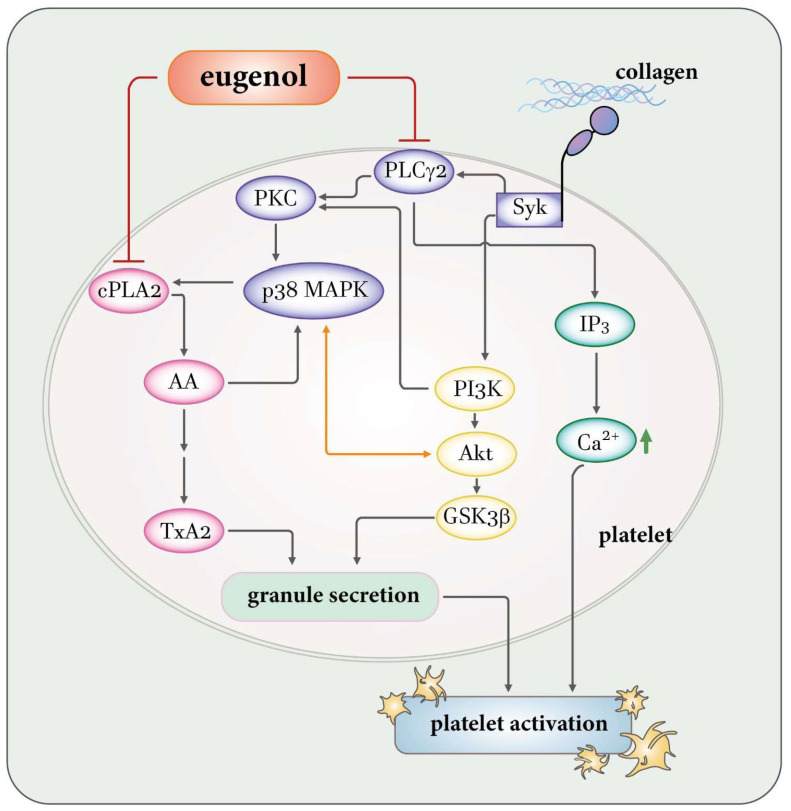
A theoretical framework provides insights into the complex mechanisms through which eugenol exerts inhibitory effects on human platelet activation. Eugenol’s impact involves targeting pivotal signaling cascades, specifically cPLA2/TxA_2_ and PLCγ2/PKC, followed by the activation of PI3K-Akt-GSK3β and MAPKs pathways. This orchestrated modulation leads to a precise control of reducing intracellular calcium ([Ca^2+^]i) levels, ultimately resulting in the suppression of platelet aggregation. In the diagram, red suppress arrows represent inhibition, orange double-head arrows signify mutual influence, and black arrows denote standard signaling pathways.

The activation of PLCγ2 is conspicuously apparent upon platelet stimulation with collagen and AA, yet remains notably absent in response to thrombin and U46619. Human platelets contain two predominant isoforms of PLC: PLCβ and PLCγ. Notably, both isoforms play distinctive roles in the signaling cascades elicited by collagen, AA, thrombin, and U46619 during platelet activation. Within the PLCγ family, isoforms 1 and 2 coexist, with PLCγ2 prominently involved in the signaling pathways instigated by collagen and AA [22,23]. Collagen, a pivotal constituent of the extracellular matrix exposed upon vascular injury, triggers platelet activation through PLCγ2-dependent pathways upon binding to specific receptors, such as glycoprotein VI (GP VI) [24]. Upon platelet activation by various stimuli, including collagen, phospholipase enzymes like cPLA_2_ are activated. These enzymes cleave AA from phospholipids in the cell membrane. Subsequently, AA can be metabolized by cyclooxygenase to generate TxA_2_, thereby amplifying platelet activation. The interconnected roles of AA-TxA_2_ and PLCγ2-PKC in platelet signaling pathways are evident, with PLCγ2 initiating signaling events and generating second messengers, while AA-TxA_2_ contributes to downstream processes enhancing platelet activation (Figure 8) [23]. Upon activation of Gαq-protein-coupled receptors (GPCRs), Gαq dissociates from the receptor and activates PLCβ, a critical step for platelet aggregation in response to most GPCR agonists like thrombin, serotonin, ADP, and TxA_2_ [25]. This elucidates why eugenol demonstrates notable efficacy in inhibiting platelet aggregation induced by collagen and AA but not by thrombin or U46619. In our study, eugenol effectively suppressed PLCγ2-PKC activation triggered by collagen. Notably, eugenol did not exert a direct influence on PKC activation, as evidenced by the unaltered platelet aggregation response induced by phorbol 12,13-dibutyrate. This intriguing observation suggests that the inhibition of PLCγ2 downstream pathways may constitute a pivotal mechanism through which eugenol exerts its inhibitory effects on platelet activation.

Platelet activation is orchestrated through intricate signaling pathways, with PI3K emerging as a pivotal contributor. PI3K assumes a critical role downstream of various platelet receptors, notably GP VI, orchestrating the activation of PLCγ2 and facilitating calcium mobilization [26]. Among the major effectors influenced by PI3K, Akt stands out, and mice lacking Akt display impaired platelet aggregation and stable adhesion under flow conditions [27]. Consequently, the PI3K-mediated activation of Akt presents a promising target for the development of antithrombotic medications. Conversely, the involvement of Akt’s downstream signaling in platelet activation remains elusive, with potential candidates such as GSK3, including its α and β isoforms, identified and expressed in platelets. Notably, GSK3β emerges as the most abundant protein among them [28]. Mice with platelet-specific PI3K deficiency manifest arterial thrombus instability under conditions of high shear stress due to impaired Akt/GSK3 activation within the developing thrombus [18]. However, the precise mechanisms through which GSK3 regulates platelet activation remain enigmatic. Therefore, the identification of GSK3’s substrates within platelets holds the potential to unveil promising targets for the development of novel antithrombotic drugs. In the realm of platelet signaling, PI3K/Akt and MAPKs undergo mutual activation, with PKC serving as the upstream regulator of MAPKs (Figure 8) [29]. Therefore, the PI3K-Akt-GSK3β signaling cascade assumes a pivotal role in platelet activation and thrombus growth and stability under conditions of high shear stress in vivo.

MAPK cascades represent indispensable signaling pathways intricately governing diverse cellular processes such as proliferation, differentiation, and apoptosis. Rigorous investigation employing MAPK-specific inhibitors and knockout mice has compellingly affirmed the involvement of ERK, JNK, and p38 MAPK in platelet activation [30]. Despite this, the precise roles of JNK and ERK in platelet activation remain enigmatic, with intriguing indications hinting at their potential as suppressors of integrin α_IIb_β_3_ activation [31]. Furthermore, the activation of ERK and JNK play a pivotal role in collagen-induced platelet aggregation [32]. The intricate interplay extends to cPLA2, playing a critical role in facilitating the release of AA to generate TxA_2_, a crucial substrate is propelled by p38 MAPK activation in response to platelet agonists (Figure 8) [32]. Our study brings to light the significant inhibitory impact of eugenol on the activation of ERK, JNK, and p38 MAPK, as well as TxA_2_ formation. This observed inhibition may elucidate the heightened efficacy of eugenol in restraining platelet activation induced by collagen or AA. Furthermore, we also conducted a fibrin clot retraction assay by introducing thrombin into a solution containing fibrinogen along with platelets treated with either 0.1% DMSO or eugenol (Figure S3). Fibrin clot retraction was more pronounced in 0.1% DMSO-treated platelets incubated for 30 min compared to those incubated for 15 min. However, fibrin clot retraction was not significantly suppressed in platelets treated with 4 μM eugenol. This observation suggests that eugenol may not interfere with platelet integrin α_IIb_β_3_ outside–in signaling.

In the exploration of the therapeutic potential of experimental compounds against vascular thrombosis, the judicious selection of animal models assumes paramount significance. Notably, the mouse model emerges as a particularly advantageous choice due to its technical simplicity, expeditious execution, and high reproducibility. Momi et al. [33] have previously demonstrated the effectiveness of this model by inducing platelet pulmonary thromboembolism in mice through the intravenous injection of collagen plus epinephrine, resulting in a dose-dependent increase in the occlusion of lung vessels by platelet thromboemboli and a significant reduction in circulating platelet numbers [33]. In alignment with these established methodologies, our current investigation similarly reveals a compelling histological observation. Following the injection of ADP, a substantially high number of lung vessels were observed to be either completely or partially occluded by platelet thrombi. This observation resonates with the recognized notion that platelet aggregation constitutes a critical risk factor for vascular thrombosis. Our study introduces a novel dimension by evaluating the therapeutic potential of eugenol, administered at a dosage of 15 mg/kg, eugenol demonstrates efficacy in reducing mortality associated with acute pulmonary thromboembolism, without concurrent alterations in bleeding time. This is in stark contrast to aspirin (15 mg/kg), a widely employed antiplatelet therapy for both primary and secondary prevention of CVDs. Intriguingly, aspirin significantly reduces the mortality rate but is accompanied by an unwanted prolongation of bleeding time. This nuanced finding positions eugenol as a promising natural compound for the treatment of thromboembolic disorders, presenting a potentially advantageous alternative to conventional antiplatelet therapies.

## 4. Materials and Methods

### 4.1. Chemicals, Reagents, and Antibodies

Eugenol (≥98.5%) was purchased from MedChem Express (Monmouth Junction, NJ, USA). Collagen (type I), aspirin, luciferin–luciferase, AA, U46619, phenylmethylsulfonyl fluoride (PMSF), sodium orthovanadate, sodium pyrophosphate, aprotinin, leupeptin, sodium fluoride (NaF), ethylenediaminetetraacetic acid (EDTA), bovine serum albumin (BSA), and thrombin were purchased from Sigma (St. Louis, MO, USA). Anti-phospho-JNK (Thr^183^/Tyr^185^), anti-phospho-PLCγ2, anti-phospho-p44/p42 ERK (Thr^202^/Tyr^204^), anti-phospho-PI3K p85 (Tyr^458^)/p55 (Tyr^199^), and anti-phospho-(Ser) PKC substrate polyclonal antibodies (pAbs) were purchased from Cell Signaling (Beverly, MA, USA). Anti-phospho-p38 MAPK (Thr^180^/Tyr^182^), phospho-cPLA2 (Ser^505^) pAbs was purchased from Affinity (Cincinnati, OH, USA). Protein assay dye reagent concentrate was purchased from Bio-Rad Laboratories Inc. (Hercules, CA, USA). Anti-phospho-Akt (Ser^473^) pAb was purchased from BioVision, Inc. (Mountain View, CA, USA). Anti-phospho-GSK3α/β and anti-α-tubulin mAbs were purchased from Santa Cruz Biotechnology (Santa Cruz, CA, USA). Fura-2-acetoxymethyl ester (Fura 2-AM) was purchased from Molecular Probes (Eugene, OR, USA). FITC-anti-human CD42P (P-selectin) mAb was obtained from BioLegend (San Diego, CA, USA). Amersham (Buckinghamshire, UK) supplied Hybond-P polyvinylidene difluoride membranes, enhanced chemiluminescence Western blotting detection reagent, horseradish peroxidase-conjugated donkey anti-rabbit immunoglobulin G (IgG), and sheep anti-mouse IgG. A 0.1% dimethyl sulfoxide (DMSO) was used to dissolve eugenol and the stock solution was stored at 4 °C. TxB_2_ enzyme-linked immunosorbent assay (ELISA) kit was purchased from Cayman Chemical (Ann Arbor, MI, USA).

### 4.2. Isolation of Human Platelets Followed by Assessment of Aggregation Capability

Approval for this study was granted by the Institutional Review Board of Taipei Medical University (TMU-JIRB-N202112047), adhering to the ethical principles delineated in the Helsinki Declaration. Informed consent was obtained from all human blood donors who participated in the study through the signing of a consent form prior to enrollment. Platelet suspensions were meticulously prepared from the blood of healthy human donors, employing a method previously outlined, which involved combining whole blood with an acid-citrate-dextrose solution (at a ratio of 9:1, *v*/*v*). Subsequent centrifugation steps were conducted to isolate platelet-rich plasma (PRP), which was then supplemented with EDTA (2 mM) and heparin (6.4 U/mL). Following a brief incubation period, another round of centrifugation was performed, and the resultant platelet pellets underwent resuspension and additional centrifugation before being suspended in Tyrode’s solution enriched with BSA at a concentration of 3.5 mg/mL and Ca^2+^ at 1 mM. Platelet counts were determined using a Coulter counter (Beckman Coulter, Miami, FL, USA). Washed platelets, adjusted to a concentration of 3.6 × 10^8^ cells/mL, were preincubated with a solvent control (0.1% DMSO) or eugenol (ranging from 0.5 to 100 μM) for a duration of 3 min before stimulation with various agonists, including collagen (1 μg/mL), AA (60 μM), thrombin (0.02 U/mL), and U46619 (1 μM). The aggregation capacity was evaluated using a lumi-aggregometer (Payton, Scarborough, ON, Canada) [34], and the extent of platelet aggregation was quantified as a percentage relative to the control group (treated with 0.1% DMSO) based on light transmission units. In the ATP release assay, luciferin-luciferase reagent was added to the platelet suspension 1 min before the introduction of collagen, and absorbance measurements were conducted using a Hitachi Spectrometer F-7000 (Tokyo, Japan) to quantitatively assess the released ATP levels.

### 4.3. Analysis of Change of [Ca^2+^]i Level and Surface Expression of P-Selectin

To assess intracellular calcium mobilization ([Ca^2+^]i), whole blood treated with citrate was centrifuged, and the resulting supernatant was incubated with 0.1% DMSO or eugenol (2 and 4 μM) and Fura 2-AM (5 μM). The levels of Fura 2-AM were measured using a Hitachi Spectrometer F-7000 (Tokyo, Japan) with excitation wavelengths of 340 nm and 380 nm, and an emission wavelength of 500 nm. In another study, the platelets were treated with eugenol (2 and 4 µM) in combination with FITC-conjugated anti-P-selectin mAb (2 µg/mL). This preincubation step lasted for 3 min. Following the preincubation, the platelets were stimulated with collagen (1 µg/mL). To analyze the platelets, a flow cytometer (FAC Scan system; Becton Dickinson, San Jose, CA, USA) was used to detect fluorescein-labeled platelets. Data were collected from 50,000 platelets per experimental group, and the platelets were identified based on their characteristic forward and orthogonal light-scattering profiles. To ensure reliability, these experiments were repeated at least four times [35].

### 4.4. Measurement of TxB_2_ Formation

Platelet suspensions (3.6 × 10^8^ cells/mL) underwent a preincubation period of 3 min with either 0.1% DMSO or eugenol (2 and 4 µM). Following this, collagen (1 µg/mL) or AA (60 µM) was introduced for 6 min. Subsequently, EDTA (2 mM) and indomethacin (500 µM) were added, and the resulting mixture was subjected to centrifugation at 2000× *g* for 5 min. Finally, TxB_2_ levels were quantified in the supernatants using an ELISA kit, adhering to the guidelines provided by the manufacturer.

### 4.5. Immunoblotting

Washed platelets (1.2 × 10^9^ cells/mL) underwent incubation with eugenol (2 and 4 μM) or 0.1% DMSO. Subsequent to this incubation, platelets were stimulated with or without collagen for 5 min. For the subsequent analytical phase, a 200 μL lysis buffer comprising aprotinin (10 μg/mL), PMSF (1 mM), leupeptin (2 μg/mL), NaF (10 mM), sodium orthovanadate (1 mM), and sodium pyrophosphate (5 mM) was introduced. The platelets were resuspended in the lysis buffer and left to incubate for 1 h. Following centrifugation at 5000× *g* for 5 min, the supernatant containing the lysates was carefully collected. From these lysates, 80 μg of protein underwent separation using 8% SDS-PAGE, and protein concentrations were determined utilizing the Bradford protein assay (Bio-Rad, Hercules, CA, USA). To facilitate the identification of specific target proteins, corresponding primary antibodies were employed for protein spot detection. The optical density of the protein bands was quantified using a video densitometer and Bio-profil Biolight software, Version V2000.01 (Vilber Lourmat, Marne-la-Vallée, France). The determination of relative protein expression involved normalizing the expression levels to the total protein content of interest.

### 4.6. Utilization of Confocal Laser Fluorescence Microscopy

Resting or collagen-activated platelets were meticulously immobilized on poly-L-lysine-coated coverslips, followed by fixation in a solution containing 4% (*v*/*v*) paraformaldehyde for 1 h. Subsequent to fixation, platelets underwent permeabilization using 0.1% Triton X-100 and were then incubated in a 5% BSA solution in phosphate-buffered saline (PBS) for 1 h to effectively block nonspecific binding sites. Following this preparatory step, platelets were subjected to immunostaining by prolonged incubation with specific primary antibodies targeting the proteins of interest over a 24-h period. Post-immunostaining, thorough washing with PBS was performed, and the platelets were subsequently exposed to secondary antibodies (Alexa Fluor^®^ 488 labeled goat anti-rabbit IgG and Alexa Fluor^®^ 647 labeled goat-anti-mouse IgG) for an additional hour. Finally, a confocal microscope (Leica TCS SP5, Mannheim, Germany) equipped with a 100× oil immersion objective was employed for imaging the platelets.

### 4.7. Acute Pulmonary Thromboembolism in Mice

Acute pulmonary microvascular thrombosis was induced in accordance with a previously delineated methodology [36]. Ethical clearance for all procedures in this investigation was secured from the Institutional Animal Care and Use Committee of Taipei Medical University (Approval ID: LAC-2022-0080). Male ICR mice were subjected to intraperitoneal injections of 50 μL of either DMSO (0.1%), aspirin (15 mg/kg) or eugenol (15 mg/kg). Following a 5-min interval, each mouse received an intravenous injection of ADP (700 mg/kg) via the tail vein. Mortality rates were meticulously recorded within 10 min post-ADP administration for each experimental group. Subsequent to extraction, the pulmonary tissues were preserved through fixation in 4% formalin, followed by embedding in paraffin. This process facilitated the generation of paraffin sections, which were subsequently subjected to hematoxylin–eosin (HE) staining. The stained lung sections underwent thorough observation, with resultant images acquired through the utilization of Microvisioneer Manual Whole Slide Imaging (manuaIWSI; Freising, Germany).

### 4.8. Tail Bleeding Time in Mice

The determination of bleeding time was conducted through the tail vein transection method. Anesthesia was induced in ICR mice via intraperitoneal injection of 50 μL of DMSO (0.1%), aspirin (15 mg/kg) or eugenol (15 mg/kg). Following a 30-min interval, the tails of the mice were precisely incised at a distance of 3 mm from the tip. The excised tails were promptly immersed in a normal saline-filled tube maintained at 37 °C for the purpose of measuring bleeding time. The duration of bleeding was recorded until the cessation of blood flow was achieved.

### 4.9. Statistical Analysis

The data are expressed as the mean ± standard error of the mean, with n denoting the number of experiments conducted using samples from distinct blood donors. To discern significant differences among the experimental groups, a one-way analysis of variance (ANOVA) was employed, complemented by the Student–Newman–Keuls post hoc test for family-wise type I error control. A predetermined threshold of statistical significance was established at *p* < 0.05. All statistical analyses were executed using SAS (version 9.2; SAS Inc., Cary, NC, USA).

## 5. Conclusions

The promotion of healthy dietary and lifestyle habits stands as a pivotal strategy for the modifiable prevention of CVDs at their earliest stages. Our investigations unveiled that eugenol exerts a potent inhibitory effect on platelet activation, achieved through the inhibition of the PLCγ2–PKC and cPLA2-TxA_2_ cascade. Consequently, this leads to a subsequent suppression of the PI3K-Akt and MAPK signaling pathways. This multifaceted action culminates in the reduction of ([Ca^2+^]i) and a consequential inhibition of platelet aggregation (Figure 8). This study underscores the potential therapeutic and prophylactic applications of eugenol in the CVDs.

## Data Availability

All data generated or analyzed during this study are included in the manuscript.

## Supplementary Materials

The following supporting information can be downloaded at: https://www.mdpi.com/article/10.3390/ijms25042098/s1.
