# Supplementary material for: Eugenol Suppresses Platelet Activation and Mitigates Pulmonary Thromboembolism in Humans and Murine Models
Source: Int J Mol Sci. 2024 Feb 8;25(4):2098. doi: 10.3390/ijms25042098 (PMC10888574; doi:10.3390/ijms25042098)
Supplement: Supplementary file 1 [file ijms-25-02098-s001.zip › ijms-2822285-supplementary.pdf]

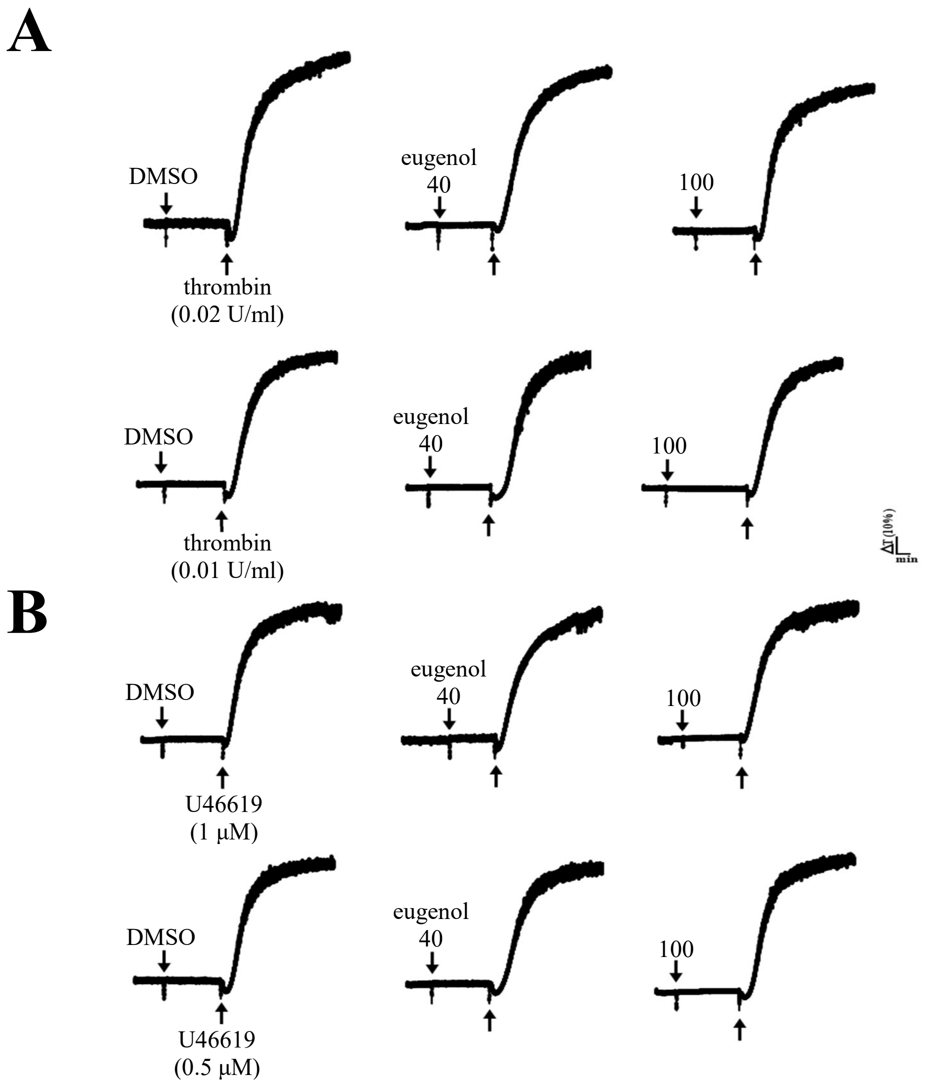

Figure S1. Washed human platelets (3.6×10^8 cells/mL) were preincubated with either a solvent control (0.1% DMSO) or concentrations of eugenol (40 or 100 μM). Subsequently, platelets were exposed to (A) thrombin (0.01 or 0.02 U/mL) and (B) U46619 (0.5 or 1 μM) to induce platelet aggregation. The concentration-response curves for eugenol emphasize its inhibitory effects on platelet aggregation induced by thrombin or U46619 (%). Profiles are representative of three similar experiments.

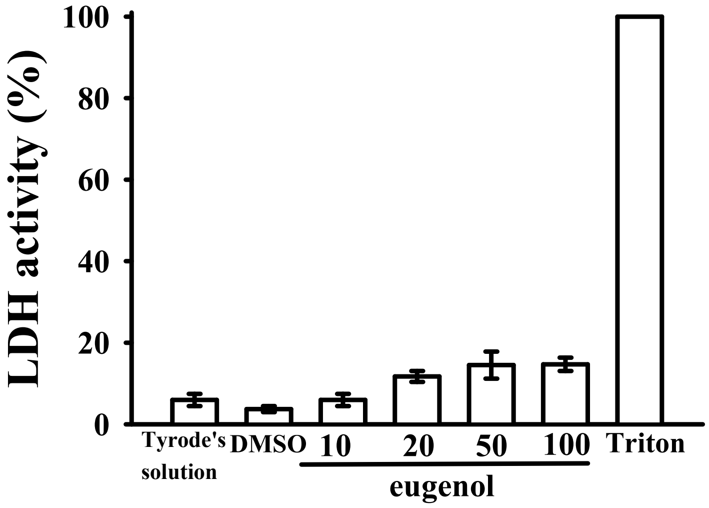

Figure S2. Effects of eugenol on platelet cytotoxicity. To assess the cytotoxicity, the solvent control (0.1% DMSO) or eugenol (10-100 μM) were pretreated in platelets for 20 min, and a 10 μL of the supernatant was dropped on a Fuji Dri-Chem slide LDH-PIII. Data are presented as the means ± standard error of the mean (n = 3).

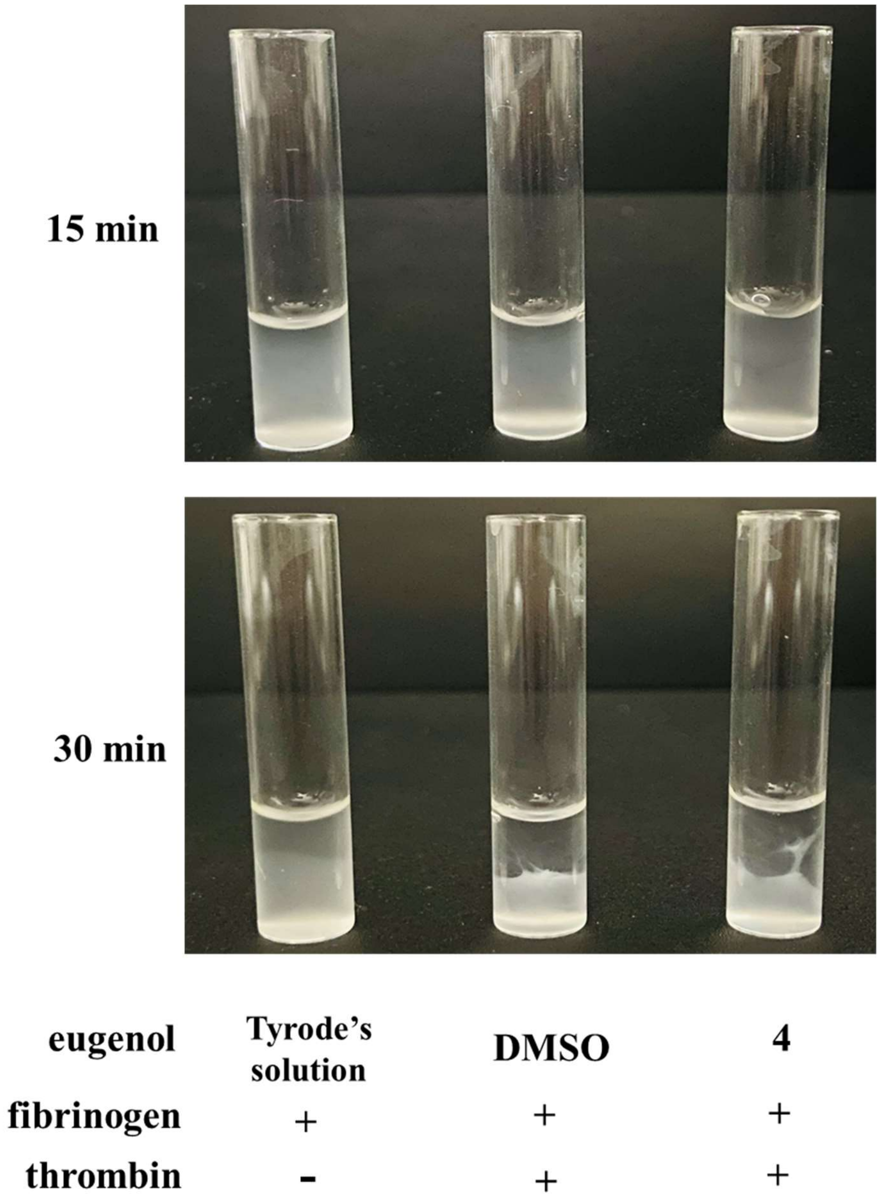

Figure S3. Effects of eugenol on clot retraction. Washed platelets (3.6×10^8 cells/mL) were suspended in Tyrode's solution containing 2 mg/mL of fibrinogen and 1 mM $CaCl_2$ with 0.1 % DMSO or eugenol (4 μM). Clot retraction was initiated with thrombin (0.02 U/mL) at 37 °C. Images were photographed at 15- and 30-min intervals by using a digital camera. Profiles are representative of three similar experiments.
